# Motivational telephone intervention to risk gamblers by a state-owned gambling operator in Sweden

**DOI:** 10.3389/fpsyt.2024.1343733

**Published:** 2024-01-30

**Authors:** Anders Hakansson, Katja Franklin, Maria Dahlström, Axel Lyckberg

**Affiliations:** ^1^Lund University, Lund, Sweden; ^2^AB Svenska Spel, Göteborg, Sweden; ^3^AB Svenska Spel, Visby, Sweden

**Keywords:** gambling disorder, behavioral addiction, motivational interviewing, problem gambling, responsible gambling

## Abstract

**Background and aims:**

Few studies have tested the effect of a motivational telephone intervention from a gambling operator to clients with high-risk gambling practices. This study aimed to study subsequent limit setting, self-exclusions and gambling post-intervention, compared to controls.

**Methods:**

The study assessed a motivational, personalized telephone intervention by the state-owned Swedish gambling operator AB Svenska Spel within its subsection of sports, poker, online casino and bingo gambling. Clients successfully reached with the telephone intervention (*n* = 1,420) were compared to clients who could not be reached (*n* = 1,504). Gambling practices during 8 weeks pre-intervention were assessed, and outcome measures limit setting, self-exclusion, and gambling 4 weeks post-intervention.

**Results:**

The telephone intervention was associated with increased limit settings (10 vs. 5 percent, *p* < 0.001), self-exclusions (11 vs. 8 percent, *p* < 0.01), lowered theoretical losses (*p* < 0.001), but not significantly associated with gambling abstinence (18 vs. 15 percent, *p* = 0.07). In unadjusted analyses of sub-groups, significant associations of the intervention with full gambling abstinence were seen in people who gamble on online casino/bingo (19 vs. 14 percent, *p* < 0.01), but not in sports bettors. In logistic regression, the intervention was not associated with full week 1–4 abstinence.

**Conclusion:**

A personalized motivational telephone intervention to people displaying high-risk gambling, delivered by a gambling operator, is promising, and effects were seen on the uptake of responsible gambling tools post-intervention. Effects may be more pronounced in users of chance-based, online games, than in sports bettors.

## Introduction

Gambling disorder is an addictive disorder characterized by extensive gambling habits, typically with a high level of loss of control, and continued gambling despite severe financial and other consequences. Gambling disorder is often associated with severe mental health consequences (1, 2).

In recent years, gambling operators’ responsibility for the detection of emerging gambling problems has been highlighted in policy work and research (3–6). This has included the overall concept of trying to identify problematic gambling behavior before these are self-reported when an individual seeks formal treatment or other measures of help (7). While the use of harm-reducing instruments may seem counter-intuitive for a gambling operator seeking to maximize profit, such responsible gambling tools have been increasingly applied by gambling operators willing to assist clients in reducing hazardous gambling as part of their responsible gambling practices. Responsible gambling tools used by gambling operators may include monitoring of gambling patterns with the aim to detect problem gambling, and may also involve the provision of harm-reducing tools such as self-exclusion, or voluntary limit setting by clients, aiming to cut down on their gambling (8). In particular, online-based gambling services technically may allow for the detection of emerging gambling problems in close temporal association with the gambling sessions, and the detection of major financial losses may constitute a window of opportunity for such interventions to take place.

Gambling problems in Sweden are reported in about 1.5 percent of the general population, with some fluctuations over the years, in nationwide survey studies defining this problem measure as a score of three or above on the problem gambling severity inventory (9). Treatment seeking for gambling disorder is in a large majority of cases based on problematic online gambling, in contrast to more traditional land-based gambling types (10, 11). Gambling advertisements seen in Swedish television are predominantly promoting online casino gambling and online sports betting (12). Treatment is available from social services of municipalities and from a limited number of health-care institutions (13).

Motivational interviewing (MI) is a model for the communication with an individual in an assessment, treatment situation or in other types of counselling or support, and includes openly framed, non-judgmental components aiming to stimulate reflection and a process or change in the individual. Key components of MI include the reflection on advantages and disadvantages of the current behavior and in a potential change, reflective listening, and the stimulation and the confirmation of change talk (14). MI has been increasingly highlighted and studied in the treatment of addictive behaviors including both substance use disorders and behavioral addictions (15). Importantly, it has been suggested that the MI approach in people with gambling problems may itself have a therapeutic effect in reducing gambling (16). For example, a brief, motivational telephone intervention has been shown to improve effects of treatment in problem gambling (17), even with a remaining effect of the intervention at a 2 years follow-up (18), or in order to improve attendance to treatment for problem gambling (19). Also, it has been highlighted that the way motivational interventions are performed, including therapist adherence to the method, is of key importance (20).

Likewise, messaging in the format of MI also has been shown to affect early stages in a help-seeking process, such as the willingness to undergo screening for gambling problems (21). Previous motivational or brief interventions have been seen from the state-owned operator assessed in the present paper, such as normative automated feedback to clients who gamble online and who display hazardous gambling patterns (22), as well as MI to people with risky gambling practices at the state-owned gambling operator of Norway (23, 24). Therefore, MI interventions can be of great interest in the responsible gambling practices of gambling operators, as part of the chain of early problem detection and secondary prevention against gambling problems.

In 2017, the state-owned gambling operator of Sweden, AB Svenska Spel, initiated a responsible gambling intervention, applying motivational, personalized telephone contact with clients with high-risk gambling patterns, with the aim of helping clients reflect upon their gambling patterns, motivate them for self-exclusion or limit-setting measures, or to seek treatment or other support services. The telephone intervention is carried out by trained officers of the gambling operator. The overall project of motivational telephone calls by AB Svenska Spel has been described in the protocol paper preceding the present results paper (25). Also, a survey study has assessed user satisfaction with the intervention. In a sample of 197 clients reached with the present intervention, during 2021 (and separate from the study reported here), a majority expressed that the telephone intervention was a favorable experience rather than the opposite, and the proportion of respondents who reported a decreasing effect on gambling, expressed as a self-reported perception, was larger than the minority who reported an increasing effect on gambling (25).

In previous studies, the delivery of a telephone intervention from a gambling operator, directly addressing clients with high-level gambling practices, was shown to be promising in the limited extent of research hitherto carried out. In an intervention from the state-owned Norwegian gambling operator, direct telephone contact proved to be more efficacious on subsequent gambling practices than a postal letter contact (23, 24). However, studies on these telephone interventions are few, and there is need for increased knowledge about their potential effects in high-risk gambling. Also, in addition to the potential for an intervention to clients with detected high-risk behaviors, it has not hitherto been demonstrated what value such responsible gambling interventions have with clients who voluntarily take a self-test, i.e., where a first step in the motivational process already can be suspected to have occurred.

In addition to the need to address this sub-group of clients identified through a self-test, the findings of Jonsson and co-workers also need to be replicated, such as in other settings where a state-owned gambling operator provides a motivational intervention as part of its responsible gambling practices. The gambling markets and policies of the Nordic countries have been characterized by large state-owned operators acting either as monopolies or as one of the operators of a larger license market, allowing for responsible gambling practices to be implemented through an operator with a business model possibly different from those of an entirely privately owned operator (26). In addition, there is reason to study the present type of intervention in settings with different types of gambling market, where the gambling types involved may differ. The project below addresses a gambling operator involved in online sports betting and online casino gambling, in a setting where these two gambling types, and particularly online casino, are strongly predominating in gambling disorder treatment settings (10, 11).

Therefore, the present study aimed to analyze, in a controlled study design, whether a motivational, personal telephone intervention to clients with high-risk gambling practices, in comparison to controls who were intended for the same intervention but who could not be reached, was associated with (1) an increase in self-limiting practices (self-exclusion and limit settings), (2) a decrease in theoretical gambling losses, and (3) an increase in gambling abstinence. Given the difficulty to specify a target level of gambling expenses estimated to be non-problematic and thereby successfully or unsuccessfully reached, as this may be variable in different clients, the gambling abstinence measure was chosen in order to add a dichotomized outcome measure over and above the changes in theoretical losses. In addition, in multi-variate analyses, the study aimed to analyze (4) potential demographic and gambling-related predictors of self-limiting practices and gambling abstinence.

## Materials and methods

The study protocol of the present study has been published (25). The data presented here refer to the quantitative controlled effectiveness study. Remaining study parts, including data describing users’ satisfaction with the intervention and post-intervention self-reported gambling data, are to be reported elsewhere.

### Setting

The present study was carried out in interventions carried out by Svenska Spel Sport & Casino, which is the sub-section of the state-owned gambling operator that provides sports betting, online poker, casino and bingo gambling. The Swedish gambling market is a license-based market since January 2019, with a high number of registered gambling operators in competitive gambling markets such as sports betting, online casino and bingo gambling, lotteries and online poker. As exceptions to this, land-based casinos (at three sites in the country) and land-based electronic gambling machines are provided as part of a state-owned monopoly. One sub-division of the state-owned gambling operator AB Svenska Spel provides the latter monopoly services, whereas one sub-division of the company provides chance-based lottery games (Svenska Spel Tur, translated into “luck”) on a competitive market, and a third sub-division provides sports- and chance-based games (Svenska Spel Sport & Casino) on a competitive market. The present study refers to the latter, i.e., the sub-division providing different types of sports betting (where bets can be made either online or in specific gambling stores, online casino, online bingo gambling and online poker). On the Swedish licensed gambling market, the registration as a client involves the identification of an individual including personal contact information (whereas for example the buying of lottery tickets in grocery stores and similar involve only an age control but no personal identification). Thus, all gambling types involved in the present study are subject to a strict identity control upon registration and upon each gambling occasion.

### Study participants

Participants were included if they fulfilled the project’s criteria of reaching above a certain level of monetary loss (more than around 9,000 Euros during the past year, or for clients younger than 25 years of age, more than around 900 Euros during 1 month), or because they were taking the self-test GamTest and coded as “red” (indicating a high risk of having a gambling problem) according to that test (27), corresponding to the level of problem described as “problem gambling” in the problem gambling severity index (PGSI) which is typically used in population studies (9). A person may be included based on a “red” self-test with or without the definition of gambling losses above. The GamTest is taken either voluntarily at the user’s own initiative as the test is displayed on the operator’s web site, or because the user may be prompted to do so as the result of detection of a hazardous gambling pattern. Whether or not a person was included by a “red” self-test was added as one co-variate in the present study.

The present study includes motivational telephone calls from the month of September, 2019, through April, 2020. Based on the ethics decision, no informed consent was obtained from study participants. All study participants were 18 years or older.

Data were available from a total of 3,562 individuals (who were included only once, or, who were included on their first attempt, provided the second attempt was at least 4 weeks beyond the first occasion). Intervention (successful call) was registered in 1,420 individuals, whereas the control condition (not reached for three contact attempts) was registered for 1,504 individuals. Further, 241 unique individuals were reached but did not want to talk, 30 responded but were unable to carry out the call, 13 were not contacted because of a recent self-exclusion activated, and in 334 individuals, adequate contact details were unavailable. In 20 individuals, no outcome of the telephone was registered. Thus, intervention (*n* = 1,420) and control subjects (*n* = 1,504) were included in the study (a total of 2,924 individuals).

### Study procedure

The intervention was defined as a motivational intervention call successfully carried out and completed, and this was compared to the control condition, which was defined to occur in individuals who were not reached despite three attempts. Occasions when the individual was reached but did not wish to talk, or when the talk could not be carried out for other (such as technical) reasons, were excluded from further study. The nature and content of the intervention calls are described in Table 1. As described there, the nature of the telephone conversation is primarily motivational and non-judgmental, and its content depends on the degree of motivation, change talk and intentions of the client. The telephone intervention starts with an introduction and the caller asks for permission to carry out the conversation, asks about the individual’s perception of her/his total gambling expenditures and gives information about her/his true amount of gambling expenditure, if allowed to do so. As one potential result, depending on the course of the conversation, information about how to limit one’s gambling limit or how to quit gambling are included. Thus, the conversation may assist subjects in self-excluding or gambling-reducing measures whenever the clients wish to, and may facilitate gambling abstinence for clients who are perceived to wish for that. Thus, the topics of self-exclusion of limit setting may not be raised in all conversations, but depending on how the conversation develops and the client’s perceived intentions. Also, the conversation is adapted to a different content for clients with obvious intention to reduce gambling. The whole conversation has the format of reflective listening aiming to stimulate the client to express ambivalence and willingness to change. The intervention is carried out by Svenska Spel staff trained in MI. All staff involved have undergone at least a 3 days MI training by a licensed psychologist experienced in gambling-related research (28), and they regularly receive updated training, as well as individual and group supervision in MI.

**Table 1:** Overall agenda in motivational calls.

Introduction of the caller and the reason for the call, and asking for permission to continue the conversation
Question about the client’s believed level of losses, and (if accepted by the client) information about her/his losses, in an attempt to create a motivational process. Information about level of losses per month. Further conversation depending on the client’s reaction to this
Attention to the client’s reaction and to motivational, change-oriented content in the client’s reaction
Open-ended questions, reflective listening. Positive feed-back to change talk detected in the client’s reaction
In case of willingness to change, active help with either setting or lowering of gambling limits, blocking increase of limits, self-exclusion, or advice about official national self-exclusion program
Assistance in creating an action plan for the client’s future gambling behavior
Summary of what has been said and agreed during the conversation

As part of the analytical strategy of the present study, the original intention was to match (in a 1:1 ratio) individuals exposed to the intervention to control individuals (25). In the present analyses, we chose not to proceed with the matching procedure, in order to limit the number of individual data lost, and in order to be able to conduct regression analyses controlling for a number of relevant variables, as it was increasingly evident that co-factors such as gambling types may need to be taken into account in regression analyses. Here, in order to be able to study the role of gambling extent for the outcomes assessed in the study, we included the level of past-28 days gambling losses as one of the predictors and one of the variables controlled for. In addition, for the main outcome in the study (gambling abstinence after the motivational call or attempts for the motivational call), this analysis was carried out separately for five different sub-segments following their level of 28 days losses. Thus, the overall strategy instead was a logistic regression analysis taking gambling losses into account, rather than a case–control procedure matching individuals based on losses. Among other changes made in the results reporting, in comparison to the original protocol paper (25), in order to limit the amount of data reported in the manuscript, detailed analyses of the time periods chosen by clients who self-exclude from gambling are not reported, and total gambling losses are also not reported (as they were considered to bring less important information than the theoretical losses measure).

The study period comprised the 8 weeks preceding the telephone call, and the 4 weeks subsequent to the call. Gambling was assessed for 8 weeks prior to the call in order to assess recent gambling patterns but while limiting the potential effects of specific, season-related events prior to the intervention. Time point zero, T0, was defined as the date of the call, or the day of the third attempt in the control group. Thus, T0 was the first day of the first week, of the four-week follow-up period (post-intervention weeks 1–4). As described below, the main analyses of the potential effect of the intervention were logistic regression analyses, in which intervention (vs. control) was included as one of the potential independent variables, along with demographic data (age, gender), whether a self-test was available (as this was one of the inclusion criteria used for the overall intervention project), the level of past 28 days losses on the Sports & Casino gambling services, and whether or not the individual had any gambling occasion registered for the pre-T0 period of the study for each of the gambling types included (pool sports betting, other sports betting, online poker, online slots gambling, other online casino gambling, or online bingo gambling). The most common gambling types used (during the eight weeks prior to T0) were pool sports betting (73 percent), other sports betting (64 percent), online casino slots (43 percent), online live casino (26 percent), and online poker (21 percent), whereas 10 percent gambling on other online casino and 7% on online bingo. In total, 56 percent (*n* = 1,651) gambled on any bingo/casino gambling, and 81 percent (*n* = 2,360) on any sports betting.

All data were derived from the client registers of AB Svenska Spel, and delivered to the first author in a fully de-identified format. The study was approved by the Swedish Ethics Authority (file number 2020/03281), and it was pre-registered on clinicaltrials.gov with the clinical trials identifier number NCT04646421 (registered 30/11/2020).

### Variables

Extent and frequency of gambling within Svenska Spel Sports & Casino

- Gambling frequency (continuous variable, days per week, 0–7 days, here calculated as the mean (M) of the 8 weeks prior to T0, and for the 4 weeks post-T0, respectively).
- Level of losses during the past 28 days prior to T0 (losses on Svenska Spel Sports & Casino gambling services, in SEK (approximately nine SEK corresponding to one USD), categorized into 2,000 SEK segments ranging from 0–1,999, 2,000–3,999, etc., through a maximum level of 40,000 and higher), and treated as a continuous variable.

Gambling types used, defined as the occurrence (or not, treated as a dichotomous variable) of any gambling registration of each of the following gambling types during any of the 8 weeks preceding T0:

- Pool sports betting games.
- Sports betting, other (online or in licensed land-based gambling stores).
- Poker games online.
- Casino slots gambling online.
- Live online casino gambling.
- Online casino, other (other chance-based casino games, including casino table games, video poker, and virtual sports betting).
- Age (in years, continuous variable).
- Gender (male, female, missing), here analyzed as a dichotomous variable describing female gender vs. male gender.
- Red self-test available or not (dichotomous variable).

Content of the telephone intervention (as summarized in the caller’s summary of the intervention call, and involving potential information/advice given after asking the client for permission to do so), each treated as a dichotomous variable (present/not present):

- Advice about limit setting was given.
- Advice for self-exclusion was given.
- Information given about gambling history.

Outcome measures post-T0:

1. Self-limiting gambling practices:
  - Self-exclusion, any time post-T0 during week 1–4, as well as time from T0 to self-exclusion (days). Any choice for self-exclusion (including self-exclusion for 1 day, 1 week, or one, three, six, 12 or 36 months, for one or all gambling types) was aggregated into one description of whether any self-exclusion had occurred, and which was analyzed here as a dichotomous variable.
  - Limit setting, any time post-T0 during week 1–4 (any limit which is either the first limit or a lowered limit compared to a previous one). Here, any limit setting carried out (including loss limits, and deposit limits, per day, week or month, respectively) was aggregated into one single measure of whether any limit had been set, and which was analyzed here as a dichotomous variable.
2. Mean weekly theoretical loss (29) post-T0 compared to pre-T0 [the wagered sum × (1-the gambling type’s return-to-player rate)], and the categorization of individuals in different magnitude of change of theoretical loss from pre-T0 (mean weekly theoretical loss during 8 weeks) to post-T0 (mean weekly theoretical loss during 4 weeks). Thus, theoretical loss was treated both as a continuous variable and categorized into discrete groups describing *a* > 50 decrease, *a* 25–50 percent decrease, *a* 0–25 percent decrease (>0 percent decrease), *a* 0–25 percent increase, *a* 25–50 percent increase, and *a* > 50 percent increase, respectively, which was treated as a Likert scale variable.
3. Gambling abstinence post-T0 (no registration of gambling on Svenska Spel Sports & Casino post-T0, week 1–4, dichotomous variable).

### Statistical methods

Group comparisons regarding categorical variables, such as comparing categorial descriptive or outcome data between the intervention and control groups, were made using the chi-square test. Corresponding group comparisons for continuous variables (age) were made using student’s *t*-test. Descriptive data were reported as number and percentages, and for continuous variables, as mean values and median values, with distribution measures of standard deviation (SD) and inter-quartile ranges (IQR), respectively.

Changes in theoretical loss from pre-T0 to post-T0 was calculated as a linear-by-linear chi-square test, testing whether there was a statistically significant trend versus a decrease in theoretical gambling loss in the intervention group, compared to the control group.

Non-stepwise logistic regression analyses were carried out for the following outcome variables: self-exclusion, limit settings, and gambling abstinence week 1–4. In these analyses, we included, as independent variables, intervention vs. control, age, gender, presence of a self-test, level of past-28 days gambling losses (categorized in 2,000 SEK sub-segments included as a continuous variable), and the occurrence (dichotomous, yes/no) of pre-T0 gambling for each of the separate gambling types. For the outcome measure of full gambling abstinence (week 1–4), separate logistic regression analyses were carried out for each gender and for individuals with any chance-based online gambling (casino gambling types and bingo) and with any type of sports betting (pool or other sports betting). In order to account for potential differences between the intervention and control groups, and in order not to leave out potential confounders among the variables studied here, we decided to include the full set of variables as independent variables in the regression analyses.

Prior to the logistic regression analyses, in order to reduce the risk of inter-collinearity, a correlation matrix was run, including all variables used as independent variables. This correlation matrix’ highest values of correlations were seen for the correlation between red self-test and level of losses (*r* = 0.43), between pool sports betting and other sports betting (*r* = 0.43), and between live casino and other casino gambling (*r* = 0.33). Other correlations were at 0.23 (live casino gambling and age) and below. None of the correlation levels demonstrated were judged to disqualify any of the variables from the regression analyses.

Due to the change in the gambling market during the spring months of 2020, due to the COVID-19 pandemic (30) (Lindner et al., 2020), a sensitivity analysis was carried out, excluding subjects included during the month of April, 2020 (391 subjects, who represented 13 percent of the full sample). In this sensitivity study, the main logistic regression analyses (gambling abstinence, self-exclusion, and limit setting) were carried out in all individuals with a T0 date from September, 2019, through March, 2020, i.e., excluding subjects with a T0 date in April, 2020. Prior to that, group comparisons were made between individuals included in April, vs. all others, in order to highlight potential differences in key variables.

All statistical calculations were made in the SPSS software (31).

## Results

A total of 2,924 individuals, who were either successfully reached (intervention) or who were called but not reached (control) were included in the study. Among them, 241 (8%) were women, 2,682 (92 percent) were men, and gender was missing for one individual. Mean age (missing in nine individuals) was 38.3 years (SD 13.9), with a median age of 36 years (range 18–79, IQR 25–49).

The mean number of weekly gambling days was 3.3 prior to T0 (SD 2.1, median 3.0, IQR 1.5–5.1), and 2.7 after T0 (SD 2.25, median 2.25, IQR 0.5–4.5). The median level of past-28 days gambling losses (available in 2,686 individuals) was 14,121 SEK (IQR 5500-31119 SEK). Self-tests were available in 506 individuals (17 percent).

Group differences in baseline characteristics between intervention and control subjects are displayed in Table 2.

**Table 2:** Clients of Svenska Spel gambling operator contacted through a motivational telephone intervention for high-risk gambling.

	Intervention (*n* = 1,420)	Control (*n* = 1,504)	*p*-value	Missing data, total
Female gender, % (*n*)	6 (86)	10 (155)	<0.001	1
Mean age, M (SD)	39.3 (13.9)	37.4 (13.8)	<0.001	9
Gambling frequency, days/week, M (SD)	3.3 (2.1)	3.3 (2.0)	0.80	0
Past-28 days losses, prior to T0 (in Swedish currency, SEK), M (SD)	23,585 (31,132)	20,485 (22,879)	<0.01	138
Self-test present, % (*n*)	17 (235)	18 (271)	0.29	
Gambling types, past 8 weeks, % (*n*)				0
Pool sports betting	73 (1,037)	72 (1,085)	0.59	
Other sports betting	62 (885)	65 (975)	0.16	
Online poker	21 (303)	21 (313)	0.73	
Online bingo	6 (92)	8 (116)	0.19	
Slots casino gambling	41 (582)	45 (680)	0.02	
Live casino	26 (367)	25 (383)	0.81	
Other casino	10 (144)	10 (157)	0.79	

Descriptive comparison of intervention group (clients successfully reached for the intervention) and control group (clients contacted but not reached, *N* = 2,924).

### Self-limiting practices (self-exclusion and limit settings)

In the intervention group, the chance of a self-exclusion was significantly higher in cases where an advice for self-exclusion was explicitly given (16 vs. 6 percent, *p* < 0.001). Also, the chance of a gambling limit set post-T0 was significantly higher in cases where advice about limit setting was explicitly delivered (13 vs. 5%, *p* < 0.001).

Self-exclusion post-intervention (9 percent, *n* = 268) was significantly more common (*p* < 0.01) in the intervention group (11 percent, *n* = 154) than in the control group (8 percent, *n* = 114). Limit setting post-intervention was seen in 7% (*n* = 213), more commonly (*p* < 0.001) in the intervention group (10 percent, *n* = 137) than in the control group (5 percent, *n* = 76).

Self-exclusion was, in logistic regression (Table 3), significantly associated with older age (*p* < 0.001), and positively associated with live casino gambling (*p* < 0.001), slots casino gambling (*p* < 0.001), and with the intervention (*p* < 0.01). Limit setting, in logistic regression (Table 4), was significantly and negatively associated with pool sports betting (*p* < 0.001), and positively associated with online casino gambling (*p* = 0.04) and with the intervention (*p* < 0.001).

**Table 3:** Logistic regression, variables in association with self-exclusion during 4 weeks after being contacted (including cases with available data on all variables, *N* = 2,679).

	OR	95 percent confidence interval	*p*-value
Age	1.02	1.01–1.03	<0.001
Female gender	1.15	0.72–1.82	0.56
Pool sports games	0.87	0.62–1.22	0.41
Sports betting	1.19	0.86–1.65	0.28
Online poker	0.99	0.70–1.39	0.93
Online casino	0.85	0.55–1.30	0.45
Online bingo	0.50	0.90–2.31	0.06
Slots gambling	3.18	2.36–4.29	<0.001
Live casino gambling	1.72	1.26–2.35	<0.001
Intervention vs. control	1.55	1.18–2.04	<0.01
Self-test	0.64	0.39–1.03	0.07
Past-28 days losses	1.01	0.99–1.03	0.37

**Table 4:** Logistic regression, variables in association with limit setting during 4 weeks after being contacted (including cases with available data on all variables, *N* = 2,679).

	OR	95 percent confidence interval	*p*-value
Age	1.00	0.99–1.01	0.81
Female gender	1.32	0.78–2.23	0.29
Pool sports games	0.90	0.62–0.30	<0.001
Sports betting	1.21	0.85–1.73	0.29
Online poker	0.81	0.55–1.19	0.29
Online casino	1.64	1.03–2.62	0.04
Online bingo	1.11	0.63–1.95	0.73
Slots gambling	1.18	0.86–1.62	0.31
Live casino gambling	0.82	0.56–1.19	0.29
Intervention vs. control	2.28	1.67–3.12	<0.001
Self-test	0.69	0.41–1.16	0.16
Past-28 days losses	1.02	1.00^*^–1.04	0.08

^*^Below 1.00, rounded off to two decimals.

### Changes in theoretical loss from gambling post-T0

The mean weekly theoretical loss in the intervention group decreased from a mean of 2,770 SEK (SD 7,378) to 2,064 SEK (SD 7,662), whereas in the control group, it decreased from 2,322 SEK (SD 5,070) to 1,879 SEK (SD 5,264). All in all, when dividing all individuals into categories of how the theoretical loss changed from pre-T0 to post-T0, there was a significant association between the intervention and being in a group presenting a decrease in theoretical gambling losses from pre-T0 to post-T0 (chi-square test, linear-by-linear, *p* < 0.001, Table 5).

**Table 5:** Changes in mean weekly theoretical loss during 4 weeks after being contacted in intervention and control subjects, respectively.

	Intervention, % (*n*)	Control, % (*n*)
>50 percent decrease	53 (762)	47 (715)
25–50 percent decrease	12 (175)	13 (192)
0^*^–25 percent decrease	10 (137)	11 (164)
0–25 percent increase	6 (87)	7 (112)
25–50 percent increase	4 (57)	5 (83)
>50 percent increase	15 (212)	17 (263)

Chi-square, linear-by-linear: *p* < 0.001. ^*^>0 percent decrease.

### Gambling abstinence post-T0

Rates of gambling abstinence during the 4 weeks post-T0 are displayed in Table 6, for all subjects and for specific sub-groups.

**Table 6:** Gambling abstinence outcome during 4 weeks after being contacted in sub-groups of included participants.

	Abstinence, intervention	Abstinence, control	*p*-value
All, % (*n*)	18 (251)	15 (229)	0.07
<10,000, % (*n*)*	20 (96)	20 (117)	0.89
10,000–19,999, % (*n*)*	16 (40)	10 (27)	0.06
20,000–29,999, % (*n*)*	12 (22)	11 (20)	0.77
30,000–39,999, % (*n*)*	12 (21)	14 (22)	0.54
>40,000, % (*n*)*	23 (46)	13 (24)	0.01
Self-test, % (*n*)	21 (50)	27 (72)	0.17
No self-test, % (*n*)	17 (201)	13 (157)	<0.01
Women, % (*n*)	21 (18)	17 (26)	0.42
Men, % (*n*)	17 (233)	15 (203)	0.09
Any pool/sports betting pre-T0, % (*n*)	13 (148)	12 (149)	0.56
Any casino/bingo gambling pre-T0, % (*n*)	19 (148)	14 (124)	<0.01
Any poker gambling pre-T0, % (*n*)	16 (48)	11 (35)	0.09

^*^Pre-T0 gambling losses, in SEK.

Gambling abstinence post-T0 was detected in 16 percent (*n* = 480). This was not significantly higher in the intervention group (18 percent, *n* = 251) than in the control group (15 percent, *n* = 229, *p* = 0.07). Post-T0 gambling abstinence was, in logistic regression (Table 7), significantly associated with younger age (*p* < 0.001) and with the presence of a self-test (*p* < 0.001), and significantly and negatively associated with pre-T0 pool sports betting (*p* < 0.001), other sports betting (*p* < 0.001), online poker gambling (*p* < 0.001), and online slots gambling (*p* < 0.01). Here, the intervention was not significantly associated with gambling abstinence (*p* = 0.08).

**Table 7:** Logistic regression, variables in association with gambling abstinence during 4 weeks after being contacted (including cases with available data on all variables, *N* = 2,924).

	OR	95 percent confidence interval	*p*-value
Age	0.98	0.97–0.99	<0.001
Female gender	0.80	0.55–1.18	0.27
Pool sports games	0.41	0.32–0.52	<0.001
Sports betting	0.44	0.34–0.56	<0.001
Online poker	0.52	0.39–0.71	<0.001
Online casino	0.96	0.65–1.40	0.82
Online bingo	0.63	0.38–1.05	0.07
Slots gambling	0.69	0.55–0.87	<0.01
Live casino gambling	1.17	0.90–1.52	0.25
Intervention vs. control	1.22	0.98–1.52	0.08
Self-test	1.81	1.33–2.44	<0.001
Past-28 days losses	1.00	0.98–1.02	0.94

Among clients with online casino/bingo gambling prior to T0, the intervention was positively and significantly associated with post-T0 gambling abstinence [OR 1.44 (1.07–1.93), *p* = 0.01], self-exclusion [OR 1.47 (1.09–1.98), *p* = 0.01], and limit setting [OR 2.05 (1.39–3.01), *p* < 0.001], when controlling for the other variables included in the logistic regression analyses. In the corresponding logistic regressions, all in sports bettors (whether or not they also gambled on chance-based gambling), the intervention was positively and significantly associated with post-T0 self-exclusion [OR 1.77 (1.29–2.43), *p* < 0.001] and with limit setting [OR 2.49 (1.75–3.53), *p* < 0.001], but not associated with gambling abstinence [OR 1.14 (0.87–1.48), *p* = 0.34].

## Discussion

The present study is one of few assessing an intervention where a gambling operator, as a part of its responsible gambling policy, carries out a personal, telephone-based, motivational interventions targeting individuals with hazardous gambling practices. When controlling for gambling types, age, gender, self-test, level of losses, the intervention was significantly associated with limit setting post-intervention and with post-intervention self-exclusions. In the whole sample, theoretical gambling losses decreased significantly after the intervention, compared to the control group. The intervention was not associated with non-gambling post-intervention. In the adjusted analyses, controlling for demographic data and for different gambling types, an independent association with gambling abstinence was seen in the highest sub-segment of gambling losses, but not in the lower ranges of gambling losses.

Altogether, in different aspects, the motivational telephone intervention described here demonstrated promising effects on key components of gambling practices. The aim of the motivational intervention is not primarily to obtain gambling abstinence unless this is perceived to be the client’s desire to obtain, but more typically introduces and reinforces a motivational process which may contribute to reduced gambling habits. Despite this, and although gambling abstinence is not necessarily an intention of the call, an effect could not be demonstrated with respect to full gambling abstinence immediately after the intervention. Here, it should be borne in mind that the intervention is not a self-selected intervention sought out by the client, but instead an intervention happening without any preparation from the user’s side, and where it must be seen as unlikely that a substantial effect would occur at once. Still, having said that, one important conclusion from the present study is that the associations with gambling abstinence were markedly more pronounced in some groups; significant and clearly larger effects were seen in the highest sub-segment of recent losses, in men, and in individuals who had any online casino/bingo gambling.

The effects of the intervention appeared to be more robust for the harm-reducing gambling tools, self-exclusion and limit setting, than for the actual outcome of gambling abstinence. Here, it can be argued that the personal motivational contact may more clearly facilitate the decision to self-exclude or limit one’s gambling, and that it was clearly less likely that these events would occur during this specific follow-up period in subjects who were not reached by the intervention. This is further strengthened by the fact that when self-exclusion or limit-setting advice was noted as a topic addressed during the conversation, this further increased the likelihood that such measures were taken, either in direct association with the call, or later.

Self-exclusion advice in the telephone conversation was associated with actual self-exclusion, and, logically, also with an increased likelihood of gambling abstinence. In contrast, it may be seen as counter-intuitive that advice about limit setting indeed was associated both with an increased likelihood of limit setting, but instead with a lower likelihood of gambling abstinence. However, it appears intuitive that a limit is chosen only by clients who choose to continue gambling to some extent, and that limit setting is unlikely in individuals who opt for a total gambling abstinence. For the same reason, information about limit setting is unlikely to be provided in a conversation with a client who actively states an intention or desire to stop gambling completely, and more likely to be provided to individuals who state an intention to gamble further. Also, it should be borne in mind that this part of the study cannot be considered to be controlled; it is likely that advice about setting limits was more likely to be delivered to people with particularly large needs for that, and where it is therefore less likely that gambling abstinence would occur. However, it can be assumed that for individuals who did set limits to their gambling post-intervention, this should have had a reducing effect on their gambling practices. The effect of the telephone intervention on limit setting was consistent with the findings from Jonsson et al. (23), where limit setting was indeed more likely to occur in individuals who had been contacted by telephone, compared to the controls and compared to the postal letter condition. In contrast, no effect on self-exclusions was seen in that study (23), but it remains likely that a motivational telephone intervention indeed has an effect on active self-limiting practices in gambling.

This study assesses a short time frame after the intervention provided. Here, it should be borne in mind that the role of this type of brief intervention is to initiate change through a motivational process, rather than to shape a more long-term therapeutic process. Thus, a client reached with the present type of brief telephone intervention may be at a very early stage in a motivational change process, or before any perception of a need to change. However, it also cannot be excluded that in some clients, the intervention delivers a relieving first contact, which may represent a type of external intervention which the individual has been sub-consciously waiting for, as part of an external control measure for an individual in a situation of severe loss of internal control. Here, also, it should be borne in mind that being recruited to the study through a “red” self-test was associated with increased likelihood of gambling abstinence, when controlling for the intervention vs. control and for a number of other co-variates. One interpretation of this may be that clients who take a self-test are already in a motivational process, and that this is an indicator of a more favorable course in the weeks following this measure. More research is needed in order to further highlight this, but the finding strengthens the rationale behind providing self-test for gambling problems to a gambling operator’s clients.

Potential effects on subsequent treatment seeking go beyond the scope of the present study. Gambling disorder is a condition which can be treated (32); different psycho-therapeutic approaches have been tested (and with hitherto less conclusive results also pharmacological strategies). Here, for example, cognitive-behavioral therapy is the treatment intervention most commonly applied. The relevance of a motivational telephone contact with clients with high-risk gambling practices can be seen in this context; a motivational conversation addressing an individual’s high-risk gambling practices may contribute to treatment seeking. It has been described that people’s treatment seeking may be diverse (33), and that it may involve either formal seeking of professional specialized treatment, or the practice of initiating online support contacts of a less formal nature, or the initiation of contacts with self-help groups as available also in the present setting. In Sweden, self-help organizations are likely to be one of the more common providers of help and support for people with gambling problems to contact (34); a Swedish web survey demonstrated that around half of respondents from the general population would advise a person with gambling problems to seek that kind of support. In addition, social services and different health-care settings are available for the assessment and treatment of problem gambling (13). Overall, based on the fact that treatment and support functions are increasingly available, this further underlines the need for motivational interventions which directly target individuals who are in close association to their high-risk gambling behaviors.

Effects differed to some extent with respect to different gambling types. However, it was also clear that associations were substantially altered in the adjusted analyses, controlling for example for the extent of gambling and demographic data, compared to the occurrence of a favorable outcome in the unadjusted analyses. Slots casino gambling and live casino gambling were the most clearly associated with self-exclusion, while such an effect was not seen for the measures describing gambling abstinence. In the unadjusted analyses, obtaining gambling abstinence was more likely in clients with any online casino/bingo gambling, where the intervention was significantly associated with abstinence, whereas in sports bettors, the overall abstinence measure was not. Thus, the study indicated a tendency towards a more extensive effect of the intervention in users of chance-based online gambling services. Online casino is more closely associated with addictive gambling in studies conducted recently in the present setting. This is particularly true when gambling practices of treatment-seeking gambling disorder patients are assessed (11), and in surveys in people who gamble online in Sweden, where online casino gambling is markedly more associated with indebtedness than online sports betting (35). Thus, a possible interpretation is that online casino gambling may be associated with a particularly addictive behaviors and that an intervention, such as the one studied here, may hold promise there in particular.

There are few studies which can be compared to the present study results, as few studies applied a direct telephone intervention specifically (6, 23, 24). Jonsson et al. (24), however, demonstrated that in some gambling sub-types, a telephone intervention was clearly superior to a letter message, and this included people with extensive online casino or online sports betting, whereas a letter invitation may be sufficient in people who display extensive patterns of lottery gambling, i.e., intuitively in gambling conditions with lower addictive potential. Thus, at least for the overall effect in clients with online casino gambling practices in the present study, this is in line with Jonsson et al. (24). Even up to 1 year post-intervention, Jonsson et al. (23) also demonstrated an increased effect on active responsible gambling measures in individuals receiving a telephone intervention compared to a control condition.

### Limitations

The present study is surrounded by certain limitations. A randomized controlled study design could not be applied here, and would require the gambling operator to randomize individuals in need for motivational support to either a motivational intervention or a non-intervention, and thereby without prior informed consent from the study subjects. Here, all individuals included were called, and subjects who were successfully reached were compared to their counterparts who—after three attempts—could not be reached. Thus, the randomization instead to a non-intervention would require either a prior consent procedure, which in itself may become a motivational intervention. However, given the intuitive need for support in individuals with highly hazardous gambling practices, it would be associated with ethical concerns if some individuals elected for the intervention would not be called. In addition, the fact that clients who are subject to the intervention are compared to individuals who did not receive this or any other intervention, may increase the difference in outcome between the groups, more than if the intervention would be compared to another active—but different—therapeutic intervention. Thus, the discrepancy between the actual intervention and the absence of intervention may be seen as a limitation (36), although at the same time, there is no golden standard procedure to compare the intervention to, as no routine for responsible gambling contacts is established. On the other hand, study participants in the intervention group are not actively seeking the intervention and are not aware of it until they receive their telephone call, and control subjects are not biases by any unmet expectations, as they are unaware of the intervention. Thus, this may reduce the limitation that this non-intervention comparison group causes.

While a randomized intervention was not possible, it can be argued that there may be group differences between individuals in the intervention group and the control group. The groups differed significantly with respect to age, gender, pre-T0 levels of gambling losses, and prevalence of online casino gambling (whereas other gambling types and other variables included did not differ significantly between groups). This fact was handled through the model where logistic regression analyses were used, including intervention vs. control as one of the independent variables, and where the factors differing between groups were also controlled for in that regression analysis.

Parts of the study period involved a gambling market affected by the COVID-19 pandemic. However, in sensitivity analyses, the exclusion of the clients called in April did not alter the main results, (when excluding clients included in April, 2020, the intervention remained significantly associated with self-exclusion, and limit setting, but not with gambling abstinence). Therefore, this is considered to be only a marginal and theoretical limitation. The brief time frame of the follow-up procedure also may be seen as a limitation. However, it should be borne in mind that the intervention studied is brief in itself, and cannot be compared for example to a formal treatment intervention. This limitation should be seen in relation to the work by Jonsson et al. (23), which carried out a follow-up measure after 1 year. Thus, future studies in the present study should aim for longer follow-up perspective. Instead, it would be of great value to study whether individuals seek treatment after the intervention or whether they take action in their gambling on other operators. Such data cannot be obtained from the present study, and requires a more qualitative study component where clients themselves report events subsequent to the intervention call.

In line with this, gambling data from other gambling operators could not be included here. It would be assumed in the study design that a motivational effect on gambling would also include a similar effect on the gambling on other operators, but such a hypothesis cannot be tested here. On the others hand, one strength of the present study is the use of actual gambling data, in relation to study designs which rely on self-reported gambling data. Studies have shown that in people who gamble online, a discrepancy often occurs between the self-perceived and self-reported level of gambling and objective, actual gambling data (37). Also, it cannot be excluded that emotional reactions to the motivational call are negative, such that the effect on an individual’s gambling on other operators would even be the opposite. Such a fear of negative reactions has been discussed, but hitherto has been shown to be limited (5, 38). One additional potential limitation is the fact that method fidelity regarding the MI conversations was not analyzed in the present study. Further study on the present motivational intervention has to include other study designs, including subjective self-report data, qualitative in-depth interviews assessing the users’ experience from the intervention, and studies assessing method fidelity in the MI-trained officers involved, which are topics not covered within the scope of the present study, and which therefore represent limitations here.

## Conclusion

An active motivational telephone intervention, carried out by a state-owned gambling operator and addressing clients with a high-risk gambling practice, appeared to have promising effects, in comparison to control subjects who were included in the intervention but who could not be reached. Effects of a motivational telephone intervention may be more distinct with respect to active harm-reducing actions such as self-exclusion or gambling limit settings, whereas potential effects on gambling abstinence may be somewhat delayed, and may not be seen when assessing the full time period after the intervention. Users of online chance-based gambling types may have larger effects from a motivational telephone intervention than sports bettors. In the whole group of individuals reached with a telephone intervention, active advisory content in the telephone call may increase the likelihood of self-exclusions or limit settings actually taking place. More in-depth studies of gambling after a motivational telephone intervention are needed, including assessments of user satisfaction and gambling outcomes in other gambling operators.

## Data availability statement

The datasets presented in this article are not readily available because according to the permissions obtained from the organisation where data were generated, and by the ethics committee, data cannot be shared publicly. Requests to access the datasets should be directed to AH, anders_c.hakansson@med.lu.se.

## Ethics statement

The studies involving humans were approved by Swedish Ethical Review Authority. The studies were conducted in accordance with the local legislation and institutional requirements. The ethics committee/institutional review board waived the requirement of written informed consent for participation from the participants or the participants’ legal guardians/next of kin because the study was conducted on retrospective data from individuals who had already been successfully reached by the intervention studied compared to individuals who were not reached.

## Author contributions

AH: Conceptualization, Formal analysis, Funding acquisition, Investigation, Methodology, Resources, Software, Supervision, Visualization, Writing – original draft. KF: Conceptualization, Data curation, Investigation, Methodology, Project administration, Resources, Supervision, Validation, Writing – review & editing. MD: Conceptualization, Investigation, Methodology, Project administration, Resources, Supervision, Validation, Writing – review & editing. AL: Conceptualization, Data curation, Investigation, Methodology, Project administration, Resources, Software, Supervision, Validation, Writing – review & editing.
